# High-throughput screening identifies candidate drugs for the treatment of recurrent respiratory papillomatosis

**DOI:** 10.1016/j.pvr.2019.100181

**Published:** 2019-08-22

**Authors:** Faris Alkhilaiwi, Siddartha Paul, Dan Zhou, Xiaohu Zhang, Feibai Wang, Nancy Palechor-Ceron, Kelli Wilson, Rajarshi Guha, Marc Ferrer, Nazaneen Grant, Craig Thomas, Richard Schlegel, Hang Yuan

**Affiliations:** a Department of Pathology, Georgetown University, Medical School, Washington DC, 20057, USA; b Department of Oncology, Georgetown University, Medical School, Washington DC, 20057, USA; c Department of Biochemistry and Molecular Biology, Georgetown University, Medical School, Washington DC, 20057, USA; d Department of Otolaryngology, Georgetown University, Medical School, Washington DC, 20057, USA; e College of Pharmacy, King Abdul Aziz University, Jeddah, Saudi Arabia; f Division of Pre-Clinical Innovation, National Center for Advancing Translational Sciences, National Institutes of Health, USA

**Keywords:** Primary cell culture, Recurrent respiratory papillomatosis, High-throughput screening, Panobinostat, Dinaciclib, Forskolin, Drug sensitivity, Repurposing

## Abstract

Recurrent respiratory papillomatosis (RRP) is a benign neoplasm of the larynx caused mainly by human papillomavirus type 6 or 11 and its standard treatment involves repeated surgical debulking of the laryngeal tumors. However, significant morbidity and occasional mortality due to multiple recurrences occur. Conditional reprogramming (CR) was used to establish a HPV-6 positive culture from an RRP patient, named GUMC-403. High-throughput screening was performed at the National Center for Advanced Technology (NCATS) to identify potential drugs to treat this rare but morbid disease. GUMC-403 cells were screened against the NPC library of >2800 approved drugs and the MIPE library of >1900 investigational drugs to identify new uses for FDA-approved drugs or drugs that have undergone significant research and development. From the two libraries, we identified a total of 13 drugs that induced significant cytotoxicity in RRP cells at IC50 values that were clinically achievable. We validated the efficacy of the drugs in vitro using CR 2D and 3D models and further refined our list of drugs to panobinostat, dinaciclib and forskolin as potential therapies for RRP patients.

## 1 Introduction

Recurrent respiratory papillomatosis (RRP) is a rare disease with an actual incidence of approximately 20,000 cases in the United States [1]. RRP is characterized by the growth of tumors in the respiratory tract caused by the human papillomavirus type 6 or 11 (HPV-6 or -11) and generally classified into two subtypes: juvenile-onset RRP (JORRP) and adult-onset RRP (AORRP) [2,3]. JORRP cases, which develop before the age of 14, are more recurrent and aggressive than their AORRP counterpart [4]. Despite the fact that RRP primarily occurs on and around the laryngeal vocal cords, these growths can spread downward and affect the bronchi, the trachea and intermittently the lung parenchyma [2,3]. Life-threatening breathing complications such as acute respiratory distress can result from untreated papilloma. When progression to the lung occurs there are limited treatment options, the disease is fatal [5].

Presently, there is no “cure” for RRP, and no single adjuvant treatment has reliably been shown to be effective in eliminating RRP [6]. The backbone of RRP therapy is surgical excision to debulk the papilloma without injuring the normal tissues [7]. A characteristic feature of this disease is the tendency for the papilloma to reappear after surgical excision, causing JORRP patients to have an average 4.4 surgeries per year [1,6,7].

One of the limitations for investigating RRP treatment has been the lack of a suitable cell culture system. As reported previously, Conditional reprogramming (CR) allows rapid and efficient isolation and propagation of primary tumor cells [8,9]. In contrast to existing conventional cancer cell lines, the conditionally reprogrammed (CR) tumor cells maintain the cancer-specific mutations and phenotypic heterogeneity typically seen in the primary tumor [[10], [11], [12]]. Therefore, CR cells represent an advanced cancer model for preclinical drug development. In the past, we isolated and propagated CR cultures from an HPV-11-positive RRP patient. We utilized the generated cell line to detect an HPV-11 mutation that may have been responsible for the observed aggressive clinical phenotype. We also used the patient's cells for a limited drug screening and identified vorinostat, an HDAC inhibitor, as a potential treatment and subsequently showed that vorinostat was effective for arresting tumor growth in the patient [13].

To extend this line of work, we have used high-throughput screening to identify potential new drugs effective against RRP. The RRP CR cells, which contained episomal HPV-6 DNA, were screened by the National Center for Advanced Translational Sciences against the NPC library of 2816 approved drugs and the MIPE library of 1912 investigational drugs. From approximately 4700 drugs we have identified 3 that might be clinically actionable.

## 2 Methods

### 2.1 Cell isolation and propagation of normal and tumor tissue samples

**Fig. 1:**
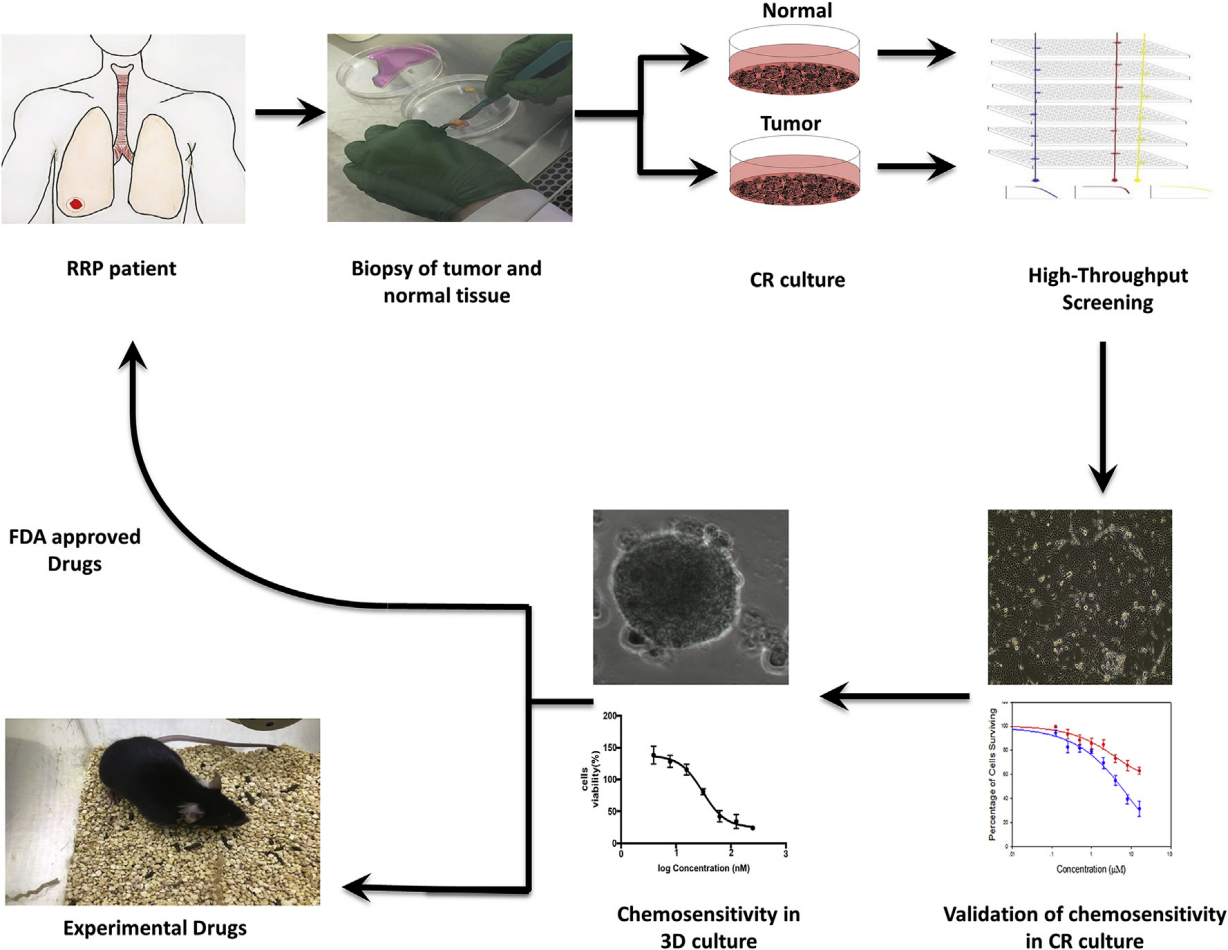
**Outline for screening conditional reprogrammed cells by High Throughput Screening.** Patient biopsies were collected from patient and examined by pathology, followed by tumor and normal cell lines establishment and propagation using Conditional Reprogramming method (CR). Next, Reprogrammed cells were tested against different drugs libraries using NCATS HTC platforms. Drugs were selected based on efficacy, safety, and specificity towards tumor cells over normal cells. In vitro 2d and 3d models were used to perform chemosensitivity tests and to select the most effective chemo agents that inhibited cell proliferation of the tumor cells. Tumor/Normal samples were collected according to the Georgetown University Institutional Review Board protocols with the informed consent of the patient.

Lung tissue (right lower lobe) was obtained at surgery with the patient's written consent according to the Georgetown University Hospital IRB. The sample was digested with collagenase and trypsin, and the obtained cells were propagated using the Conditionally Reprogrammed (CR) system (Fig. 1). that includes the use of a bed of irradiated murine fibroblasts and a F medium supplemented with Y-27632 Rho-kinase inhibitor (Enzo Life Sciences) as described previously [8,9]. The cells, named as GUMC-403, were used for HTS, 2D and 3D cell viability assays. Previously a laryngeal sample from another patient was used to generate normal cells, named GUMC-228 (HPV negative), which used as a control in the 2D cell viability assay.

### 2.2 DNA isolation, cloning, and sequencing

DNA was isolated and purified from the cultured cells, GUMC-403, or directly from the tissues using the DNeasy Blood and Tissue Kit (Qiagen) and was amplified with specific primers for HPV-6, HPV-11 or general primers for HPVs or with the Rolling Circle Amplification (RCA) kit (Illustra TempliPhi, GE Healthcare Life Sciences), as published previously [13]. The products of RCA were digested with EcoR I, BamHI, Hind III, Nde I and EcoR V. Two DNA fragments from the digestion were isolated from agarose gel, and cloned into pUC19 separately and sequenced from two directions with the use of Primer Walking Services (Genewiz).

### 2.3 HPV-6 viral copy number assay and E6:E2 ratio assay

Genomic DNA was extracted from RLL-403 and GUMC-403 and diluted with nuclease-free water to 10 μg/ml then used for all ddPCR experiments. ddPCR, probes were designed to amplify products of 100-300bp as recommended. The reaction mixtures contained ddPCR Probe Supermix (Bio-Rad Laboratories, Hercules, CA, USA), template DNA (1 μl) and primers (final concentration, 25 nM) in a final volume of 20 μl. Each reaction was then loaded into a sample well of an eight-well disposable cartridge (DG8™; Bio-Rad Laboratories) along with 70 μl of droplet generation oil (Bio-Rad Laboratories). A QX200™ Droplet Generator (Bio-Rad Laboratories) was used to generate the droplets as per the manufacturer's directions. Droplets were then transferred to a 96-well PCR plate, heat-sealed with foil and amplified using a conventional thermal cycler to the end point (95 °C × 5 min (1 cycle), 95 °C × 30 s, 56 °C × 30 s and 72 °C × 30 s (40 cycles), followed by 4 °C × 5 min (1 cycle), 90 °C × 5 min (1 cycle), and lastly 4 °C hold). A QX200 Droplet Reader (Bio-Rad Laboratories) was used to read the resulting products, and QuantaSoft™ software (Bio-Rad Laboratories) to analyze data. Thresholds bar was set based on the value of the no template control (NTC), and the copy number was calculated based on the ratio of HPV-6 L1: RNaseP. RNaseP copy number reference assays were purchased commercially (Applied Biosystems). The HPV6-L1 Tagman assay used is forward primer 5′-(TGG AAG ATG TAG TTA CGG ATG C) -3′, reverse primer 5′-(AGC CCA GGG ACA TAA CAA TG) -3′, and probe 56-FAM/AC CAC ACG C/ZEN/A GTA CCA ACA TGA CA/3IABkFQ. A E2/E6 ratio assay specific for HPV-6 was adopted from an earlier study [14]. The HPV6-E6 Tagman assay used is forward primer 5′-(CCACGTCTGCAACGACCATA) -3′, (reverse primer) 5′-(TTCCATGAAATTCTAGGCAGCA) -3′, and probe FAM-5′- CCTG TTTCGAGGCGGCTATCCA-3′-TAMRA.The HPV6-E2 Tagman assay used forward primer 5′-(AAAAGTATGGGAGCACCAAACA-) -3′, (reverse primer) 5′-(GCTGGTCGTGATTGTTAGTGATG) -3′, and probe FAM-5′-TGGACCCGTGGACAGTGGAAACC-3′-TAMRA. The same protocol was followed for E6: E2 ratio assay except for an annealing temperature of 61 °C × 30 s.

### 2.4 Amplification of papillomavirus oncogene transcripts PCR (APOT-PCR) for HPV-6

For detection of the physical status of HPV6 (episomal vs. integrated), a 3′-RACE APOT assay was used which was based on Huebbers et al. [15]. After reverse transcription of RNA, a nested PCR with a set of 5′-Primers (1st 5′-primer: 5′-GGACGGACAAGATTCACAACC-3′; 2nd 5′-primer: 5′-CCTGTTGCTGTGGATGTGACAGC-3′) both located in the E7 open reading frame of HPV-6 and a 3′-Frohman primer (for both nested PCR-setups) was used. PCR products were separated on a 1% agarose gel. Visible bands were cut out of the gel, purified (Gel extraction kit, QIAGEN) and sequenced. Sequences were compared with NCBI and UCSC database entries to determine viral sequences or virus-human fusion sequences indicating viral integration.

### 2.5 Chemotaxis/cell migration assay

Cell migration experiments were performed using the xCELLigence RTCA (ACEA Biosciences Inc.) system as described before [16]. xCELLigence allows the examination of the cell migration process in real time by measuring electrical impedance. Experiments were carried out in 16-well plates (CIM-16, ACEA Biosciences Inc.). Briefly, 160 μL of cell culture medium with and without serum were dispensed in the lower chambers while 50 μL serum-free medium were added to the upper chamber followed 1 h incubation. Next, the background was read. The experiment was paused and the plate removed from the RTCA-DP device. Then, 1.0 × 10^5^ of GUMC-403 cells or Human Foreskin Keratinocytes (HFK) cells in a total volume of 50-μL serum-free medium/well were seeded on the upper chamber. Plate was placed back in the RTCA system and incubated for 27 h, performing measurements every 10 min. The electrical impedance is reflection of cell number and software was used to generate the migration activity of each condition. The graph represents the average of triplicates.

### 2.6 High throughput screen (HTS) assay

GUMC-403 cells were plated at a density of 700 cells/well in 5 μL of (F + Y medium) into white tissue culture treated polystyrene plates (Corning Cat. 7464) using a MultiDrop Combi dispenser with small volume cassette. All plates were covered with a stainless steel gasketed lid and placed into an incubator at 37 °C/95% RH/5% CO_2_ overnight. The next day, 23 nL of MIPE 4.0 library, NPC library, and control compounds were added to each plate using a pin tool dispenser [17,18]. Controls included bortezomib at 9.2 μM, DMSO only, and empty wells. Plates were then returned to the incubator for 48 h. To assess cell viability, 3 μL of CellTiter-Glo reagent (Promega) was added to each well of the plates using a solenoid value dispenser. Plates were then incubated at room temperature for 15 min and then, luminescence signal was read using a ViewLux (PerkinElmer) with a 2 s exposure time. Data normalization was done using DMSO as 100% cell viability and empty wells as 0% viability.

### 2.7 Cell viability assay for 2D and 3D

GUMC-403 cells (5.0 × 10^3^ cells/well in 100 μl of F + Y medium) were seeded in a 96 wells plate for 2D monolayer (VWR, Radnor, PA) or (5.0 × 10^3^ cells/well in 50ul of F + Y medium) in a 96-well ULA round-bottomed plates for 3D spheres culture (CLS3474, corning). Seeded cells were incubated overnight at 37 °C in a cell culture incubator with 5% CO2 levels. For 2D drug culture treatments, the medium was replaced with fresh medium that have 7 different concentrations of the 13 drugs (drugs are listed in Table 1). For the 3D drug culture treatments, 7 different concentrations of Panobinostat, Dinaciclib or Forskolin were added in 50 ul to make up the volume to 100 ul. Cell viability assay was conducted using The Veritas microplate luminometer turner Biosystems. The CellTiter-Glo® Luminescent Cell Viability Assay (G7570, Promega, Madison, WI) kit for 2D culture or CellTiter-Glo® 3D Cell Viability Assay (G9681, Promega, Madison, WI) kit for 3D culture and GloMax®-96 Microplate Luminometer Software (Promega) were used for data analysis according to the manufacturer's protocol. The cell viability reading was measured after 3 days for 2D culture or 5 days for 3D culture and the treated cells luminescence reading was normalized to that of vehicle (DMSO) treated cells. For statistical significance, the experiment including was carried in a 3 technical replicates and conducted at three independent times.

**Table 1:** The best 13 drugs from NCATS screening.

Drugs	Name	Mode of Action	Diseases Treated	Library
Drug 1	Panobinostat	HDAC inhibitor	Multiple myeloma(approved),HIV-HAART combination (in trial)	MIPE
Drug 2	Dinaciclib	CDK2, CDK5, CDK1 and CDK9 inhibitor	In clinical trials for various cancer	MIPE
Drug 3	Forskolin	ubiquitous activator of eukaryotic adenylyl cyclase	Glaucoma	NPC
Drug 4	Verteporfin	Photo-sensitizing agent derived from porphyrin in endothelial cells.	Photodynamic therapy for abnormal blood vessels	MIPE
Drug 5	Fomepizole	Inhibitor of the enzyme alcohol dehydrogenase.	Antidote for methanol or ethyl alcohol poisoning	MIPE
Drug 6	Carlfilzomib	Selective proteasome inhibitor	Multiple myeloma	MIPE & NPC
Drug 7	Flavopiridol	Inhibitor of CDKs including CDK1, CDK2, CDK4 and CDK6	Acute myeloid Leukemia	MIPE & NPC
Drug 8	AT-7519	Multi CDK inhibitor	Metastatic Tumors, Multiple myeloma	MIPE
Drug 9	SNS-032	CDK inhibitor	Chronic lymphocytic leukemia. (In phase I trial)	MIPE
Drug 10	Romidepsin	HDAC1 and HDAC2 inhibitor	Cutaneous T-cell Lymphoma (CTCL)	MIPE
Drug 11	PF-04691502	PI3K(α/β/δ/γ)/mTOR dual inhibitor	Solid Tumors	MIPE
Drug 12	Sertindole	5-HT_2_ serotonin and D_2_ dopamine receptor antagonist and antipsychotic.	Antipsychotic in schizophrenia	NPC
Drug 13	Crenolanib	selective inhibitor of PDGFRα/β, and FLT3 inhibitor	In clinical trials for leukemia, glioma, NSLCC	MIPE

### 2.8 Xenograft assay

To determine in vivo tumorigenicity for the GUMC-403, 1 × 10^6^ cells were suspended in 200 μL of Matrigel HC (BD-growing Biosciences). The Matrigel-suspended cells were injected subcutaneously into the left and right flanks of 6-week-old male mice with severe combined immunodeficiency (Taconic, Germantown, NY). The growth of xenografts was measured weekly with callipers. Animals were housed at the Georgetown University animal care facility according to institutional guidelines. Animal protocol #14-033-100171 was approved by Institutional Animal Care and Use Committee (IACUC) at Georgetown University. All experiments were performed in accordance with the protocol relevant guidelines and regulations.

### 2.9 Statistical analysis

Unpaired student's t-test was used to compare drug treatment response in primary cells. Data (mean ± s.e.m.) were calculated and plotted using GraphPad Prism 6.0 (La Jolla, CA).

## 3 Results

### 3.1 Generation and characterization of HPV-6 positive CR cultures from RRP patient

**Fig. 2:**
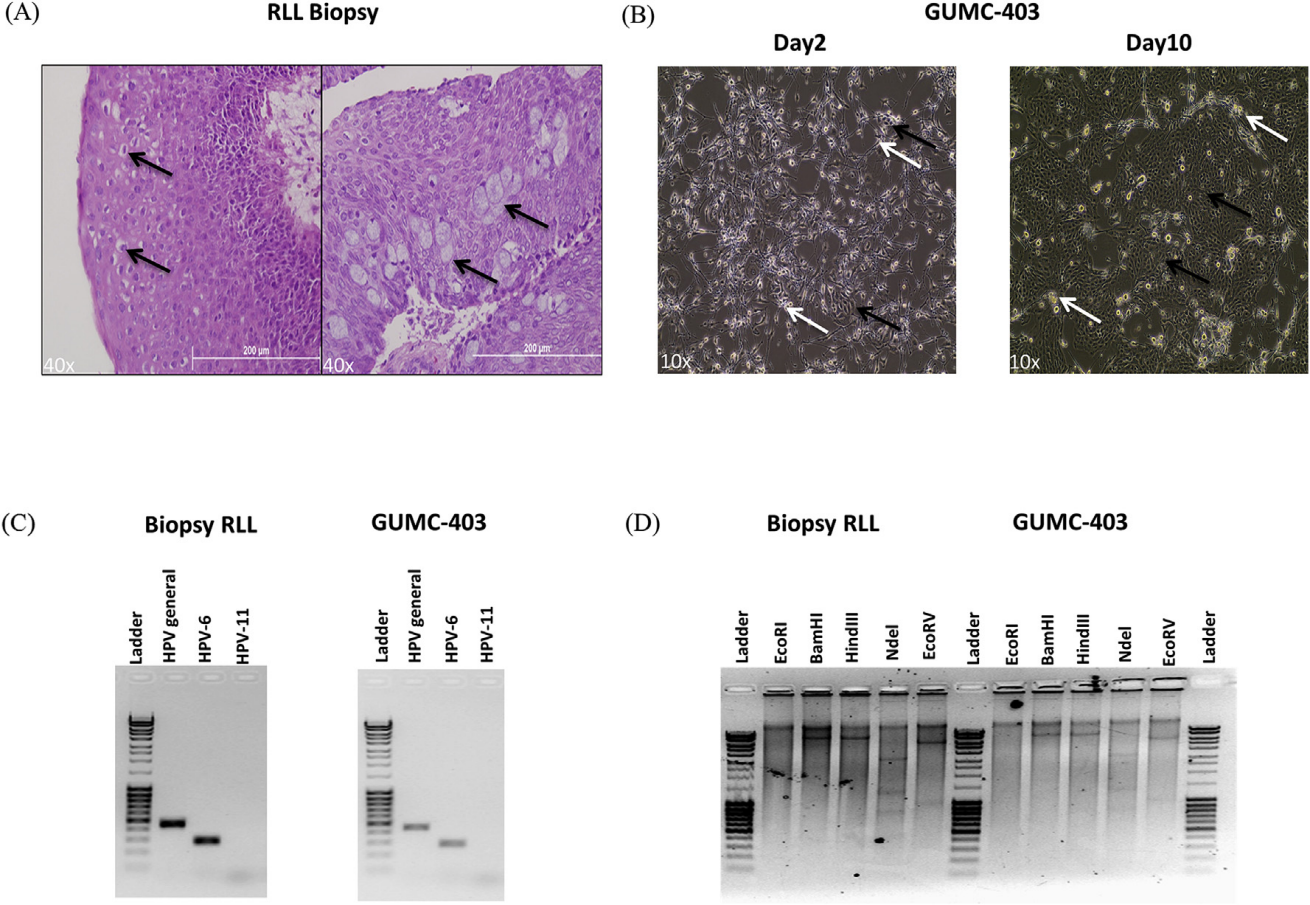
**Generation and HPV typing of conditionally reprogrammed Recurrent Respiratory Papillomatosis (RRP) cells from a neoplastic lung biopsy.** (A) Lung biopsy H&E staining is displaying koilocytotic atypia (Left, black arrow) and intraepithelial mucocytes (Right, black arrow). (B) Phase contrast images of GUMC-403 (black arrow) cocultured with irradiated feeders (white arrow) on day 2 (Left) and day 10 (Right). (C) Genotyping with HPV-6 and HPV-11 primers showing PCR Results indicates the exclusive presence of HPV-6 DNA in RLL biopsy and GUMC-403 samples. (D) Enzyme-restriction pattern using EcoRI, BamHI, HindIII, Ndel, and EcoRV in the HPV-6 genome after rolling circle amplification, HPV-6 from RLL biopsy and GUMC-403 show one band with BamHI, one band with HindIII, three bands with Ndel and two bands with EcoRV suggesting the episomal nature of the viral DNA. (40× magnification. Size bars = 200 μm in (A) and 10× magnification in (B)).

The patient was a 29 year old female with more than a 26-year history of recurrent respiratory papillomatosis. She had undergone more than 90 laryngeal ablation surgeries to control viral-induced tumors and had been additionally treated with intralesional cidofovir. However, the treatment was not able to slow tumor growth or its progression into the lung. Computed Tomographic (CT) scanning revealed that there were multiple pulmonary nodules that had accelerated in growth. Bronchoscopy was performed and papillomas from the right lower lobe (RLL) were excised and submitted for both pathologic examination and cell isolation. Histology of the tumor revealed squamous papillary proliferation (Fig. 2A, Left) and intraepithelial mucocytes (Fig. 2A, Right). To facilitate the analysis of molecular alterations and drug screening in RRP, we established a cell culture (GUMC-403) from a right lower lobe papilloma biopsy using conditional reprogramming [9]. The cells could be observed as early as 2 days after isolation, and the primary culture reached confluence in 10 days (Fig. 2B). The cells were maintained for more than 28 population doublings (52 days) with an average growth rate of 45 h/doubling.

HPV typing using specific primers for HPV-6 or HPV-11 or general primers for HPVs was conducted. Only bands with primers for HPV-6 and general HPV were detected in the biopsy and GUMC-403 (Fig. 2C). The amplified bands were isolated and cloned. Sequencing of the PCR products showed that all the products matched HPV-6 DNA. HPVs can exist in two forms in the infected cells either episomal form or integrated form [19]. To confirm the anticipated episomal form of the HPV-6 DNA in GUMC-403, we performed rolling circle amplification (RCA) followed by digestion by a set of restriction enzymes [20]. The expected number and size of bands for HPV-6 were detected in the biopsy and GUMC-403 (Fig. 2D). HPV-6 from the lung tissue and GUMC-403 cells exhibited no bands with EcoR I enzyme, one band (8000 kb) with BamHI, one band (8000 kb) with Hind III, 3 bands (3200 kb, 1300 kb, 800 kb) with NdeI and 2 bands (6000 kb, 1000 kb) with EcoR V. There were two assays established in previous studies looking for viral integration of HPV6. The 3′ rapid amplification of cDNA ends (RACE) amplification of papillomavirus oncogene transcripts (APOT) assay specific for HPV6 described previously by Huebbers et al. was performed to detect the physical status of viral genome [15]. The 3′-RACE PCR allows differentiation between episome- and integrate-derived E6/E7 mRNA transcripts. Using this assay, we only found viral mRNAs, and no host-viral fused RNAs were detected. We also use E2/E6 ratio to investigate HPV integration status. An E2/E6 ratio assay specific for HPV-6 was established in a previous study [14]. E2/E6 ratio about 1 was regarded as “episome state,” whereas ratio of 0 was considered as “fully integrated state”. Our results indicated E2/E6 ratios as 1.12 in the RRP biopsy and 1.06 in our CR cells. This suggested that most viral HPV6 genomes remained as episomal. This agreed with earlier studies result that low risk HPVs rarely integrate into human genomes. To determine if the HPV-6 genome might contain significant mutations, the whole viral genome was cloned and sequenced. Unlike in our previously reported RRP case, we did not detect any mutations in the viral genome [13]. This cell line represents an HPV-6 positive cell culture containing episomal viral DNA.

Quantatative PCR was used for viral copy number assessment. The average viral copy number is 1.08 copy per cell in GUMC-403 at passage 2. Viral genome copy number gradually decreased during passaging, and was undetectable at passage 6. This is consistent with earlier studies that these HPV positive cells lack the maintenance of the eipsomal viral genome. All the characterization assays and drug-screening experiments were done between passages 2 and 3. Quantitative PCR assays were performed to verify the presence of HPV-6 genome at the time.

### 3.2 GUMC-403 cells maintain RRP characteristics

**Fig. 3:**
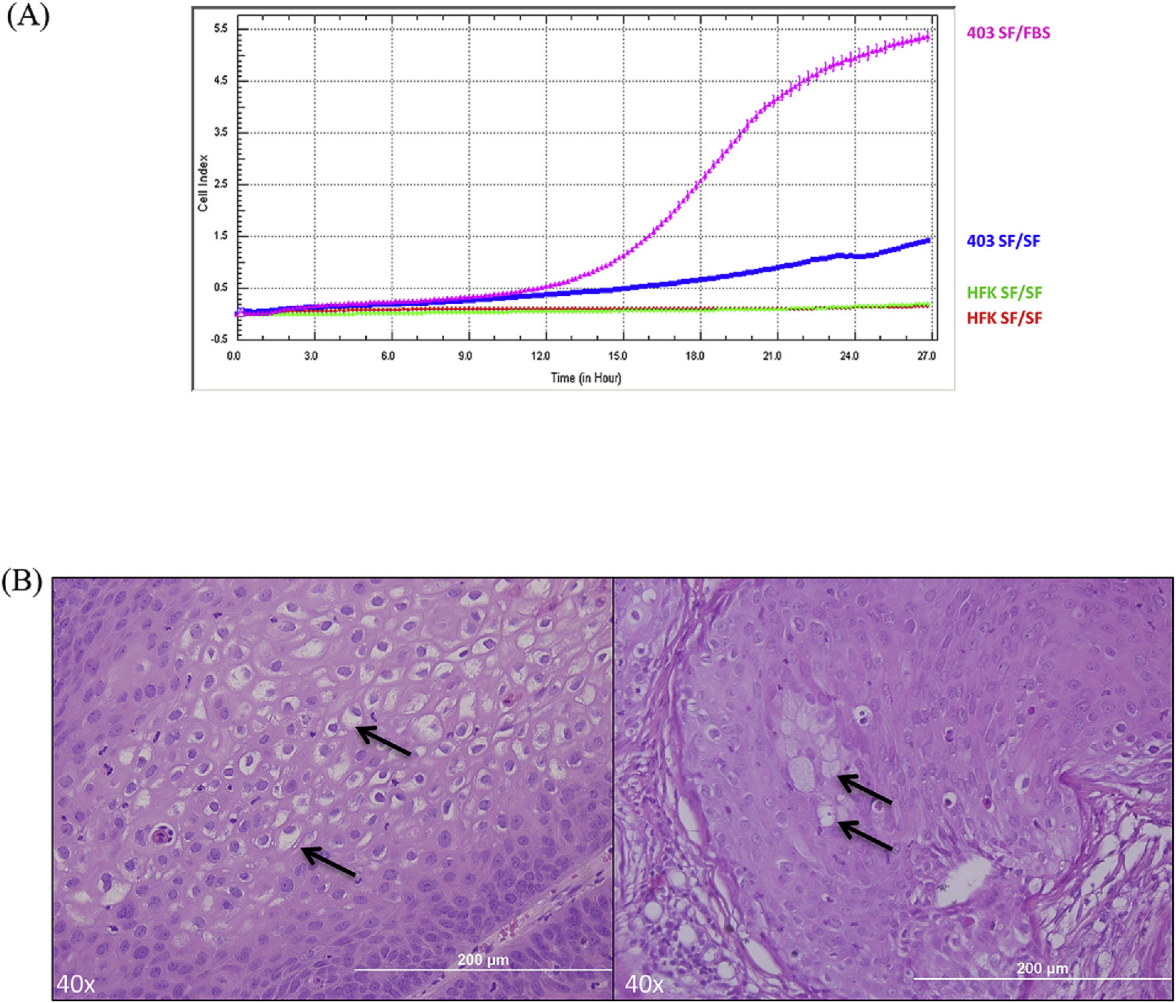
**GUMC-403 cells maintain invasive and tumorigenic properties.** (A) GUMC-403 cells exhibit a higher migration capacity in either the presence or absence of FBS compared with HFK (The graph is the average of triplicates). (B) Xenograft H&E staining is displaying koilocytes (Left, black arrow) and intraepithelial mucocytes (Right, black arrow). (40× magnification. Size bars = 200 μm).

The capacity of tumor cells to migrate is a critical property for cancer metastasis [21]. Therefore, a transwell migration assay was used to measure the chemotaxis of GUMC-403 cells by electric impedance in response to a chemoattractant fetal bovine serum (FBS). GUMC-403 cells showed higher migration potential compared to human foreskin keratinocytes (HFK, negative control) in the presence or in the absence of fetal bovine serum (FBS) during a 27 h period (Fig. 3A). We further analyze GUMC-403 for in vivo tumorigenicity, cells were trypsinized from a culture at passage 5 and injected subcutaneously into immunodeficient mice. The tumors were measurable as early as 8 weeks post-injection. Xenograft experiments were performed three times independently, with a total of 11 out 12 xenograft sites producing tumors. Similar to the primary tumor (Fig. 2A), the xenografts were composed of atypia squamous papillary proliferation with focal koilocytotic (Fig. 3B, Left) and intraepithelial mucocytes (Fig. 3B, Right). These results demonstrate that the cell line GUMC-403 maintains the tumorigenic phenotype in vitro and in vivo and mimic the original tumor.

### 3.3 High Throughput Screening assay for RRP cells

Drug screening was performed at the National Center for Advancing Translational Sciences (NCATS) using 1536 well plates. Drugs from the NPC library (8 point dilutions, 1:5 dilution) and MIPE library (11 point dilutions, 1:3 dilution) were evaluated at concentrations ranging from 0.5 nM to 50 μM^15,16,^. Survival curves with an area under the curve (AUC) less than 425 for MIPE or less than 460 for NPC and Curve Class of −1.1 were selected [22]. Out of 4728 drugs, 45 drugs matched the criteria, and 13 were selected for further validation in 2D and 3D cultures. Priority was given to compounds based on their degree of cell killing, clinical status and FDA approval.

### 3.4 2D and 3D drug sensitivity validation

**Fig. 4:**
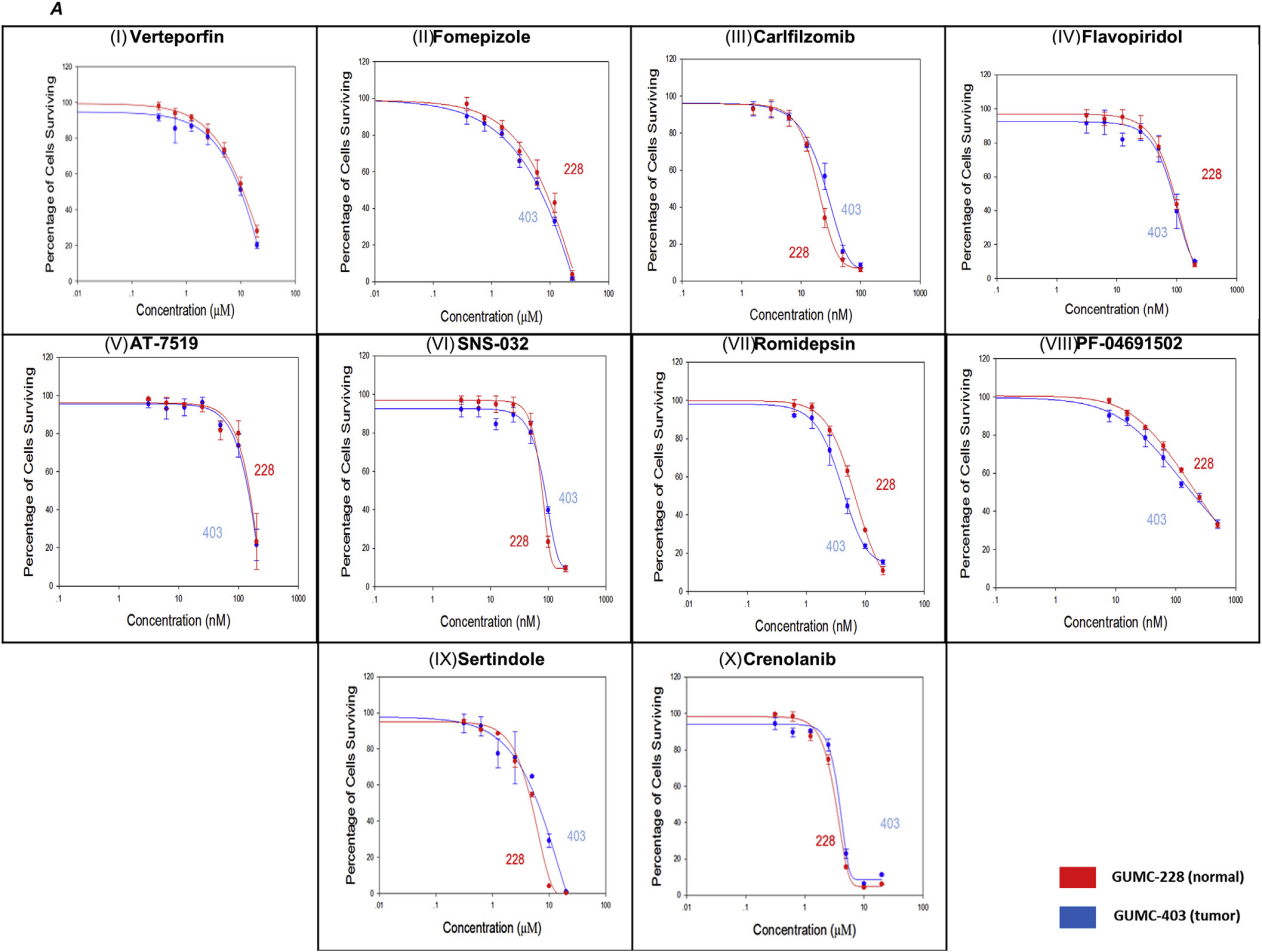

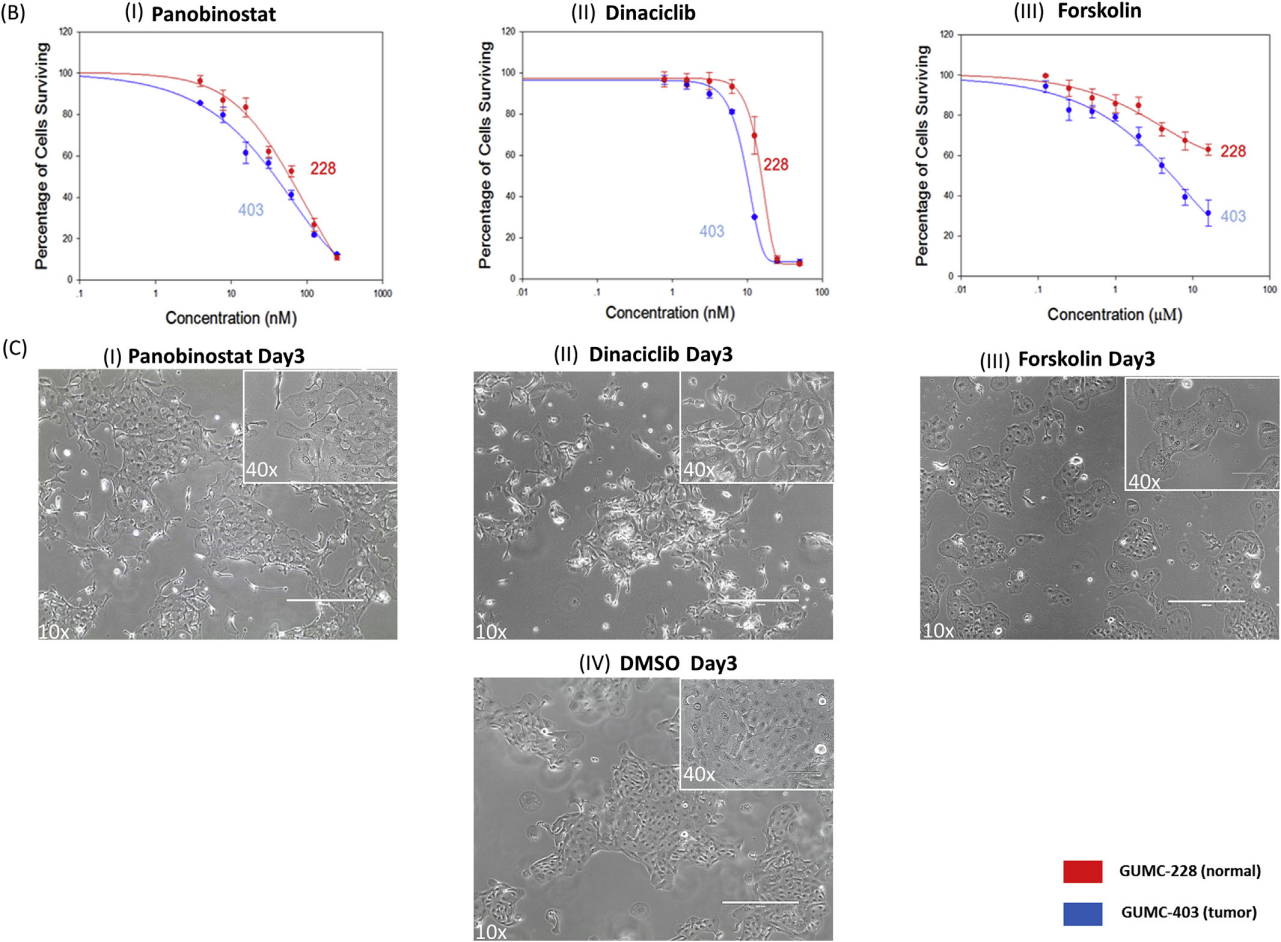
**Validation of drugs cytotoxicity on 2D culture of GUMC-403 and GUMC-228.** (**A)** Dose–response curves for (I) Verteporfin, (II) Fomepizole, (III) Carlfilzomib, (IV) Flavopiridol, (V) AT-7519, (VI) SNS-032, (VII) Romidepsin, (VIII) PF-04691502, (IX) Sertindole, (X) Crenolanib. (B) Dose–response curves for (I) Panobinostat, (II) Dinaciclib and (III) Forskolin show differential toxicity toward GUMC-403 over GUMC-228 (P value is 0.0014, 0.0018 and 0.000005 for Panobinostat, Dinaciclib and Forskolin respectively). (C) Phase contrast images of GUMC-403 cells treated with (I) Panobinostat, (II) Dinaciclib (III) Forskolin show evidence of abnormal stressed cells compared to the vehicle control (IV) DMSO treated cells (right) (10× magnification. Size bars = 400 μm). Top right images show enlarged magnification (40× magnification, size bars = 100 μm). Data represents mean ± s.d. from 3 independent measurements, each in triplicate. Unpaired student T test was performed on the data and *p* value < 0.05 was considered statistically significant.

The 13 potential candidates from NCASTS screening (Table 1) were further validated using 2 and 3-dimensional culture systems. For the validation assays and to further narrow down the potential candidates, we used normal laryngeal cells isolated from a second RRP patient, named GUMC-228 (HPV negative), since the GUMC-403 patient did not provide normal tissue. 5.0 × 10^3^ cells/well of GUMC-403 and GUMC-228 were seeded in 96 well plates, allowed to attach for 24 h. The cell monolayer was then treated for 72 h with (Fig. 4A,I) Verteporfin (Fig. 4A,II), Fomepizole (Fig. 4A,III), Carlfilzomib (Fig. 4A,IV), Flavopiridol (Fig. 4A,V), AT-7519 (Fig. 4A,VI), SNS-032 (Fig. 4A,VII), Romidepsin (Fig. 4A,VIII), PF-04691502 (Fig. 4A,IX), Sertindole and (Fig. 4A,X) Crenolanib in concentrations ranging from 50 uM to .5 nM. The cell viability assays revealed higher selective cytotoxicity toward GUMC-403 compared with GUMC-228 when treated with (Fig. 4B,I) Panobinostat (Fig. 4B, II), Dinaciclib and (Fig. 4B, III) Forskolin, depicted by an IC50 of 0.035 uM for Panobinostat, 0.010 uM for Dinaciclib and 5.157 uM for Forskolin (Table 2). The Cytotoxic effect of these drugs induced morphological changes within 3 days of treatment and ultimately a decreased cell viability compared to DMSO treated control (Fig. 4C I, II, III and IV).

**Table 2:** IC50 comparison between NCATS and CR 2D, 3D chemosensitivity.

No.	Drugs	NCATS IC50 (μM) 2D	CRC center IC50 (μM) 2D	CRC center IC50 (μM) 3D
1	Panobinostat	2.600	0.035	.0304
2	Dinaciclib	0.046	0.010	.0104
3	Forskolin	1.670	5.157	1.920
4	Verteporfin	0.660	8.898	
5	Fomepizole	0.330	5.406	
6	Carlfilzomib	1.180	0.024	
7	Flavopiridol	0.130	0.079	
8	AT-7519	1.040	0.133	
9	SNS-032	0.460	0.083	
10	Romidepsin	.1040	0.005	
11	PF-04691502	0.265	0.188	
12	Sertindole	2.600	5.460	
13	Crenolanib	0.008	3.674	

**Fig. 5:**
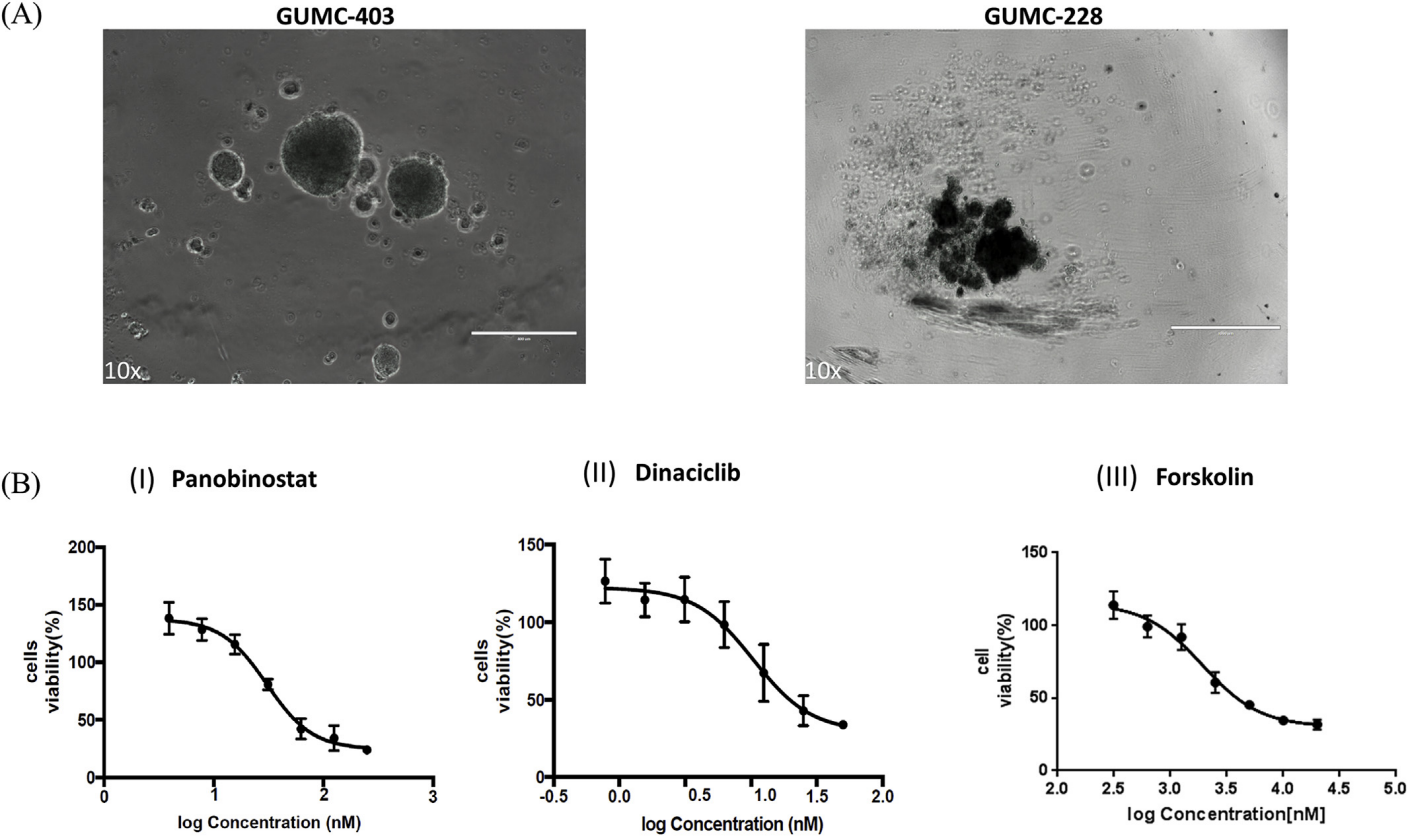
**Validation of cytotoxicity of Panobinostat, Dinaciclib and Forskolin on the GUMC-403 3D culture.** (A) Morphology of GUMC-403 spheres in ULA round-bottomed plates (left) and GUMC-228 (right). (B) Dose–response curves for (I) Panobinostat, (II) Dinaciclib and (III) Forskolin show similar cytotoxicity to 2D on 3D culture of GUMC-403. (10× magnification. Size bars = 400 μm). Data represents mean ± s.d. from 3 independent measurements, each in triplicate.

In recent years, the use of 3D cell culture systems has been increasingly used as an in vitro model for drug discovery. Therefore testing drug candidates for efficacy and tissue distribution can be enhanced through 3D culture such as multicellular tumor spheroids (MCTS) due to the in vivo like microenvironment [23]. 5.0 × 10^3^ cells/well of GUMC-403 and GUMC-228 were seeded in low attachment plates and incubated overnight. GUMC-403 cells formed spheres around the sizes of 200–400 uM (Fig. 5A left) while GUMC-228 cells failed to form spheres (Fig. 5A right). The RRP cells were treated for 5 days with Panobinostat, Dinaciclib or Forskolin. In the 3D culture, the IC50 of Panobinostat, Dinaciclib or Forskolin were 0.030 uM, 0.010 uM and 1.920 uM respectively, as calculated from dose-response curves (Fig. 5B, Table .2). Thus, we found that Panobinostat, Dinaciclib, and Forskolin have a similar cytotoxicity for RRP Cells in 2D and 3D in vitro models.

## 4 Discussion

Recurrent respiratory papillomatosis is a fatal disease once it has metastasized to the lung and there are limited therapeutic options since no single adjuvant therapy has been shown to be effective in eliminating RRP. A major limitation to study RRP tumor progression and treatment is the lack of an appropriate cell culture system. Recently CR culture has shown to maintain and imitate the ordinary biology of their primary tissue such as tracheal epithelium, ectocervical epithelium or breast tumors [10,11]. Additionally, intra-tumoral heterogeneity was maintained in CR cells suggesting oligoclonality of these cultures [12]. In an earlier study, we isolated and established a continuous CR cell cultures from a patient with recurrent respiratory papillomatosis. The HPV-11 positive CR cell culture helped us to detect a unique and important mutation in the viral genome. More importantly, the primary patient's RRP cells enabled us to run a limited-scale drug screening and identified an effective therapy for the patient in less than two weeks [13]. In this study, we successfully established a RRP cell culture system that contains episomal HPV-6. Unlike in the earlier HPV-11 positive RRP cells, we did not detect any significant mutations of the HPV6 viral genome in this cell line. In xenograft assays, measurable tumors were observed as early as eight-weeks post injection into immunodeficient mice. The original cellular characteristics of the RRP tumor, as well as HPV genome, were maintained in the xenografts.

Recently, CR cells have been used in translational research for drug discovery. Using small scaled high-throughput drug screening with 306 clinical and emerging cancer drugs on CR cells, Saeed et al. (2017) have identified Bcl-2 family inhibitor navitoclax as a potential treatment for castration-resistant prostate cancer (currently is being tested in a clinical trial) [24]. Chen et al. (2017) have used CR to grow cells from rare salivary gland cancers and identify regorafenib as a potential therapeutic drug [25]. Recently, Alamri et al. (2018) have shown that allosteric AKT inhibitor MK2206 can inhibit the growth of Mucoepidermoid carcinoma (MEC) cells in 2d and 3d CR culture [26]. Formerly, we have used CR method to identify vorinostat as an effective treatment for an HPV-11 positive RRP case; the appropriate therapy was identified in less than two weeks [13]. In contrast of the limited, small-scale drug screening in our earlier HPV-11 case using 96-well plates, the drug repurposing study in this HPV6 case was done in a high throughput format with thousands of drugs using 1536-well plates. The rapid expansion of 35 million cells of the RRP CR cells met the demand for a large number of testing cells.

Drug repurposing has emerged a novel approach in finding new treatments for unmet health conditions due to the well-defined side-effect profiles and the established bioavailabilities of drugs that led to FDA approval [27,28]. In this study, we identified three drugs cytotoxic to RRP cells and all of them have IC 50s that are within the safe range of therapeutic use and covering different classes of drugs such as CDK inhibitors, HDAC inhibitors, and non cancer drugs such as Forskolin. Panobinostat has been approved for multiple myeloma and cutaneous T-cell lymphoma [29,30]. Additionally, panobinostat (Farydac®, LBH589) is under clinical investigation for a range of hematological and solid tumors worldwide in both oral and intravenous formulations [29,31]. Panobinostat represses tumor cell growth by interacting with nonhistone and histones proteins as well as autophagy-mediated targets, apoptotic and tumorigenesis pathways involved in the development of cancer [32]. Recently, Vorinostat and Panobinostat were shown to inhibit HPV-18 E6 and E7 activities, leading to stabilize host cell tumor suppressors as well as abolishing the viral DNA amplification [33].

Our drug screening also found CDK inhibitors that had potent cytotoxicity against RRP cells. For instance, dinaciclib is an inhibitor of CDK1, CDK2, CDK5, and CDK9, and is active in a broad range of cancer cell lines originating from leukemia, melanoma, osteosarcoma and pancreatic cancer [[34], [35], [36], [37]]. The mechanism by which dinaciclib inhibits RRP cells is unknown but previous study has shown that it inhibits RB phosphorylation in cancer cells at concentrations between 12 and 500 nM [38,39]. The IC50 from this study is 10 nm from 2D and 3D cell validation assays. Interestingly, the combination of HDAC and CDK inhibitors is a new leukemic and melanoma strategy since in combination they activate caspase; induce mitochondrial damage, and alter cell cycle regulation [40,41]. Therefore, we will explore the potential synergy of HADCi and CDKi present for RRP treatment.

Unlike panobinostat and dinaciclib, forskolin is a natural product that has been isolated from the roots of the plant Coleus Forskohlii [42]. Forskolin exhibits a wide range of pharmacological properties such as anti-obesity, asthma, and glaucoma by the stimulation of adenylyl cyclase activity and increases intracellular levels of cyclic AMP [[43], [44], [45]]. It has also been reported forskolin has anti-cancer activity. Treatment of colon cancer line KM12C with the adenylyl cyclase activator Forskolin completely inhibits their growth at the concentration of 50uM [46,47]. In the present study, we demonstrated that forskolin was cytotoxic to RRP cells at concentrations as low as 1.67 uM.

The use of three-dimensional (3D) cellular systems for drug discovery has been explored in drug discovery. A recent study, using the same HTS screen platform, showed similar drug responses between cancer cell lines growing as 2D monolayers and 3D spheres [48]. In this study, we developed the first 3D tumor model of RRP using patient's primary CR cells. Interestingly, we were able to validate drug sensibility in our 3D sphere system, and IC50s for the three drugs were similar in 2D and 3D systems. However, the 3D RRP culture does have the potential to discover novel mechanisms and targets and to accelerate target identification and validation, given that the gene expression patterns found in 3D models can better mimic physiological conditions [23].

Early studies have shown that low-risk HPV is known to be difficult to maintain in cultured cells [49]. In GUMC-403 cells, the average viral copy number is 1.08 copy per cell at passage 2. Viral genome copy number gradually decreased during passaging, and was undetectable at passage 6. All the characterization assays and drug-screening experiments were done between passages 2 and 3. Quantitative PCR assays were performed to verify the presence of HPV-6 genome at the time. Interestingly the xenograft assay with a relative late passage cells (passage 5) produced 11 tumors out of the 12 xenograft sites. More importantly the xenografts were composed of atypia squamous papillary proliferation with focal koilocytotic and intraepithelial mucocytes. These results suggested that even in the late passage the cell line GUMC-403 maintains the tumorigenic phenotype. CR cells, like GUMC-403, can be very useful for drug screening assay for RRP. However, the importance of validation of the HPV presence as well as the maintenance of tumorigenic phenotype should be performed at the same time.

## 5 Conclusion

In this study, by using conditional reprogramming and high throughput screen platforms, we identified and validate Panobinostat, Dinaciclib or Forskolin as effective drugs for recurrent respiratory papillomatosis therapy.

## Funding

This work was funded by internal grant support (H.Y. and F.A) from the Center for Cell Reprogramming at GUMC and grant support (C.T.) from the division of Preclinical Innovation of the National Center for Advancing Translational Sciences.

## Author contributions

Conception and design: F.A., S·P., F·W., H.Y. and R.S. Development of methodology: F.A., F·W., D.Z., S·P., X.Z.,K·W., R.G., M.F., C.T and H.Y. Performed the high-throughput assay:., X.Z., R.G., M.F., C.T. Acquisition of data: F.A., F·W., D.Z., S·P., X.Z.,K·W., R.G., M.F., C.T and H.Y. Analysis and interpretation of data: F.A., D.Z., F·W., K·W.,L.A., H.Y. and R.S.; Writing, Review and/or revision of the manuscript: F.A., H·Y and R.S.; Administrative, technical, or material Support: F.A., D.Z., S·P., N.G., and H.Y. Study supervision: F.A., H·Y., T.C., and R.S.

## Conflicts of interest

The authors declare no competing financial interests.

## List of abbreviations

CR: Conditional Reprogramming
PCR: polymerase chain reaction
3D: three-dimensional
2D: two-dimensional
ROCK: Rho-associated protein kinase
HFK: human foreskin keratinocyte;
PDs: population doublings.

## References

[1] Armstrong L.R., Derkay C.S., Reeves W.C. (1999). Initial results from the national registry for juvenile-onset recurrent respiratory papillomatosis. RRP Task Force. Arch. Otolaryngol. Head Neck Surg..

[2] Fortes H.R., von Ranke F.M., Escuissato D.L., Araujo Neto C.A., Zanetti G., Hochhegger B., Souza C.A., Marchiori E. (2017). Recurrent respiratory papillomatosis: a state-of-the-art review. Respir. Med..

[3] Dyrstad S.W., a Rao K. (2008). Recurrent respiratory papillomatosis (RRP)-Juvenile onset. Clin. Med. Oncol..

[4] Derkay C.S. (1995). Task force on recurrent respiratory papillomas: a preliminary report. Arch. Otolaryngol. Head Neck Surg..

[5] Goon P., Sonnex C., Jani P., Stanley M., Sudhoff H. (2008). Recurrent respiratory papillomatosis: an overview of current thinking and treatment. Eur. Arch. Oto-Rhino-Laryngol..

[6] Derkay C.S., Wiatrak B. (2008). Recurrent respiratory papillomatosis: a review. The Laryngoscope.

[7] Rodman R., Mutasa S., Dupuis C., Spratt H., Underbrink M. (2014). Genetic dysregulation in recurrent respiratory papillomatosis. The Laryngoscope.

[8] Liu X., Ory V., Chapman S., Yuan H., Albanese C., Kallakury B., Timofeeva O.A., Nealon C., Dakic A., Simic V., Haddad B.R., Rhim J.S., Dritschilo A., Riegel A., McBride A., Schlegel R. (2012). ROCK inhibitor and feeder cells induce the conditional reprogramming of epithelial cells. Am. J. Pathol..

[9] Liu X., Krawczyk E., Suprynowicz F.A., Palechor-Ceron N., Yuan H., Dakic A., Simic V., Zheng Y.-L., Sripadhan P., Chen C., Lu J., Hou T.-W., Choudhury S., Kallakury B., Tang D., Darling T., Thangapazham R., Timofeeva O., Dritschilo A., Randell S.H., Albanese C., Agarwal S., Schlegel R. (2017). Conditional reprogramming and long-term expansion of normal and tumor cells from human biospecimens. Nat. Protoc..

[10] Mahajan A.S., Sugita B.M., Duttargi A.N., Saenz F., Krawczyk E., McCutcheon J.N., Fonseca A.S., Kallakury B., Pohlmann P., Gusev Y., Cavalli L.R. (2017). Genomic comparison of early-passage conditionally reprogrammed breast cancer cells to their corresponding primary tumors. PLoS One.

[11] Suprynowicz F.A., Upadhyay G., Krawczyk E., Kramer S.C., Hebert J.D., Liu X., Yuan H., Cheluvaraju C., Clapp P.W., Boucher R.C., Kamonjoh C.M., Randell S.H., Schlegel R. (2012). Conditionally reprogrammed cells represent a stem-like state of adult epithelial cells. Proc. Natl. Acad. Sci..

[12] Correa B.R.S., Hu J., Penalva L.O.F., Schlegel R., Rimm D.L., Galante P.A.F., Agarwal S. (2018). Patient-derived conditionally reprogrammed cells maintain intra-tumor genetic heterogeneity. Sci. Rep..

[13] Yuan H., Myers S., Wang J., Zhou D., Woo J.A., Kallakury B., Ju A., Bazylewicz M., Carter Y.M., Albanese C., Grant N., Shad A., Dritschilo A., Liu X., Schlegel R. (2012). Use of reprogrammed cells to identify therapy for respiratory papillomatosis. N. Engl. J. Med..

[14] Coutlée F., Trottier H., Gagnon S., Koushik A., Richardson H., Roger M., Ferenczy A.S., Franco E.L. (2009). Low-risk human papillomavirus type 6 DNA load and integration in cervical samples from women with squamous intraepithelial lesions. J. Clin. Virol..

[15] Huebbers C.U., Preuss S.F., Kolligs J., Vent J., Stenner M., Wieland U., Silling S., Drebber U., Speel E.J.M., Klussmann J.P. (2013). Integration of HPV6 and downregulation of AKR1C3 expression mark malignant transformation in a patient with juvenile-onset laryngeal papillomatosis. PLoS One.

[16] Celik H., Hong S.-H., Colon-Lopez D.D., Han J., Kont Y.S., Minas T.Z., Swift M., Paige M., Glasgow E., Toretsky J.A., Bosch J., Uren A. (2015). Identification of novel ezrin inhibitors targeting metastatic osteosarcoma by screening open access malaria box. Mol. Cancer Ther..

[17] Huang R., Southall N., Wang Y., Yasgar A., Shinn P., Jadhav A., Nguyen D.T., Austin C.P. (2011). The NCGC pharmaceutical collection: a comprehensive resource of clinically approved drugs enabling repurposing and chemical genomics. Sci. Transl. Med..

[18] Heske C.M., Davis M.I., Baumgart J.T., Wilson K., Gormally M.V., Chen L., Zhang X., Ceribelli M., Duveau D.Y., Guha R., Ferrer M., Arnaldez F.I., Ji J., Tran H.L., Zhang Y., Mendoza A., Helman L.J., Thomas C.J. (2017). Matrix screen identifies synergistic combination of PARP inhibitors and nicotinamide phosphoribosyltransferase (NAMPT) inhibitors in Ewing sarcoma. Clin. Cancer Res..

[19] Williams V.M., Filippova M., Soto U., Duerksen-Hughes P.J. (2011). HPV-DNA integration and carcinogenesis: putative roles for inflammation and oxidative stress. Future Virol..

[20] Kusumoto-Matsuo R., Kanda T., Kukimoto I. (2011). Rolling circle replication of human papillomavirus type 16 DNA in epithelial cell extracts. Genes Cells.

[21] Hall A. (2009). The cytoskeleton and cancer. Cancer Metastasis Rev..

[22] Inglese J., Auld D.S., Jadhav A., Johnson R.L., Simeonov A., Yasgar A., Zheng W., Austin C.P. (2006). Quantitative high-throughput screening: a titration-based approach that efficiently identifies biological activities in large chemical libraries. Proc. Natl. Acad. Sci..

[23] Friedrich J., Seidel C., Ebner R., Kunz-Schughart L.A. (2009). Spheroid-based drug screen: considerations and practical approach. Nat. Protoc..

[24] Saeed K., Rahkama V., Eldfors S., Bychkov D., Mpindi J.P., Yadav B., Paavolainen L., Aittokallio T., Heckman C., Wennerberg K., Peehl D.M., Horvath P., Mirtti T., Rannikko A., Kallioniemi O., Östling P., af Hällström T.M. (2017). Comprehensive drug testing of patient-derived conditionally reprogrammed cells from castration-resistant prostate cancer. Eur. Urol..

[25] Chen C., Choudhury S., Wangsa D., Lescott C.J., Wilkins D.J., Sripadhan P., Liu X., Wangsa D., Ried T., Moskaluk C., Wick M.J., Glasgow E., Schlegel R., Agarwal S. (2017). A multiplex preclinical model for adenoid cystic carcinoma of the salivary gland identifies regorafenib as a potential therapeutic drug. Sci. Rep..

[26] Alamri A.M., Liu X., Blancato J.K., Haddad B.R., Wang W., Zhong X., Choudhary S., Krawczyk E., Kallakury B.V., Davidson B.J., Furth P.A. (2018). Expanding primary cells from mucoepidermoid and other salivary gland neoplasms for genetic and chemosensitivity testing. Dis. Model.Mech..

[27] Ashburn T.T., Thor K.B. (2004). Drug repositioning: identifying and developing new uses for existing drugs. Nat. Rev. Drug Discov..

[28] Li Y.Y., Jones S.J.M. (2012). Drug repositioning for personalized medicine. Genome Med..

[29] U.S.F. (2015). D. administration (FDA), press announcements - FDA approves farydak for treatment of multiple myeloma, U.S. Department of health and human Services. https://www.fda.gov/NewsEvents/Newsroom/PressAnnouncements/ucm435296.htm%0Ahttp://www.fda.gov/NewsEvents/Newsroom/PressAnnouncements/ucm435296.htm.

[30] Vandermolen K.M., McCulloch W., Pearce C.J., Oberlies N.H. (2011). Romidepsin (Istodax, NSC 630176, FR901228, FK228, depsipeptide): a natural product recently approved for cutaneous T-cell lymphoma. J. Antibiot..

[31] Jones S.F., Bendell J.C., Infante J.R., Spigel D.R., Thompson D.S., a Yardley D., Greco F.A., Murphy P.B., a Burris H. (2011). A phase I study of panobinostat in combination with gemcitabine in the treatment of solid tumors. Clin. Adv. Hematol. Oncol.: HO (Hum. Organ.).

[32] Singh A., Patel P., Jageshwar, Patel V.K., Jain D.K., Kamal M., Rajak H. (2017). The safety, efficacy and therapeutic potential of histone deacetylase inhibitors with special reference to panobinostat in gastrointestinal tumors: a review of preclinical and clinical studies. Curr. Cancer Drug Targets.

[33] Banerjee N., Moore D., Broker T., Chow L. (2018). Vorinostat, a pan-HDAC inhibitor, abrogates productive HPV-18 DNA amplification. Proc. Natl. Acad. Sci..

[34] Johnson A.J., Yeh Y.Y., Smith L.L., Wagner A.J., Hessler J., Gupta S., Flynn J., Jones J., Zhang X., Bannerji R., Grever M.R., Byrd J.C. (2012). The novel cyclin-dependent kinase inhibitor dinaciclib (SCH727965) promotes apoptosis and abrogates microenvironmental cytokine protection in chronic lymphocytic leukemia cells. Leukemia.

[35] Desai B.M., Villanueva J., Nguyen T.T.K., Lioni M., Xiao M., Kong J., Krepler C., Vultur A., Flaherty K.T., Nathanson K.L., Smalley K.S.M., Herlyn M. (2013). The anti-melanoma activity of dinaciclib, a cyclin-dependent kinase inhibitor, is dependent on p53 signaling. PLoS One.

[36] Fu W., Ma L., Chu B., Wang X., Bui M.M., Gemmer J., Altiok S., Pledger W.J. (2011). The cyclin-dependent kinase inhibitor SCH 727965 (dinacliclib) induces the apoptosis of osteosarcoma cells. Mol. Cancer Ther..

[37] Feldmann G., Mishra A., Bisht S., Karikari C., Garrido-Laguna I., Rasheed Z., Ottenhof N.A., Dadon T., Alvarez H., Fendrich V., Rajeshkumar N.V., Matsui W., Brossart P., Hidalgo M., Bannerji R., Maitra A., Nelkin B.D. (2011). Cyclin-dependent kinase inhibitor dinaciclib (SCH727965) inhibits pancreatic cancer growth and progression in murine xenograft models. Cancer Biol. Ther..

[38] Knudsen E.S., Wang J.Y.J. (2010). Targeting the RB-pathway in cancer therapy. Clin. Cancer Res..

[39] Whittaker S.R., Mallinger A., Workman P., Clarke P.A. (2017). Inhibitors of cyclin-dependent kinases as cancer therapeutics. Pharmacol. Ther..

[40] Almenara J., Rosato R., Grant S. (2002). Synergistic induction of mitochondrial damage and apoptosis in human leukemia cells by flavopiridol and the histone deacetylase inhibitor suberoylanilide hydroxamic acid (SAHA). Leukemia.

[41] Heijkants R., Willekens K., Schoonderwoerd M., Teunisse A., Nieveen M., Radaelli E., Hawinkels L., Marine J.-C., Jochemsen A. (2018). Combined inhibition of CDK and HDAC as a promising therapeutic strategy for both cutaneous and uveal metastatic melanoma. Oncotarget.

[42] Shen Y.H., Xu Y.L. (2005). Two new diterpenoids from coleus forskohlii. J. Asian Nat. Prod. Res..

[43] Godard M.P., Johnson B.A., Richmond S.R. (2005). Body composition and hormonal adaptations associated with forskolin consumption in overweight and obese men. Obes. Res..

[44] González-Sánchez R., Trujillo X., Trujillo-Hernández B., Vásquez C., Huerta M., Elizalde A. (2006). Forskolin versus sodium cromoglycate for prevention of asthma attacks: a single-blinded clinical trial. J. Int. Med. Res..

[45] Majeed M., Nagabhushanam K., Natarajan S., Vaidyanathan P., Karri S.K., Jose J.A. (2015). Efficacy and safety of 1% forskolin eye drops in open angle glaucoma - an open label study. Saudi.Journal of Ophthalmology.

[46] Agarwal K.C., Parks R.E. (1983). Forskolin: a potential antimetastatic agent. Int. J. Cancer.

[47] McEwan D.G., Brunton V.G., Baillie G.S., Leslie N.R., Houslay M.D., Framee M.C. (2007). Chemoresistant KM12C colon cancer cells are addicted to low cyclic AMP levels in a phosphodiesterase 4-regulated compartment via effects on phosphoinositide 3-kinase. Cancer Res..

[48] Mathews Griner L.A., Zhang X., Guha R., McKnight C., Goldlust I.S., Lal-Nag M., Wilson K., Michael S., Titus S., Shinn P., Thomas C.J., Ferrer M. (2016). Large-scale pharmacological profiling of 3D tumor models of cancer cells. Cell Death Dis..

[49] DiLorenzo T., Taichman L., Steinberg B. (1992). Replication and persistence of HPV DNA in cultured cells derived from laryngeal papillomas. Virology.

